# Executives’ academic experience and corporate social responsibility: A case study in China

**DOI:** 10.1371/journal.pone.0305813

**Published:** 2024-06-25

**Authors:** XiFeng Yang, MeiHui Qi

**Affiliations:** 1 Department of International Education, Jiangxi Science and Technology Normal University, Nanchan, Jiangxi Province, China; 2 Department of Business Administration, Lingnan Normal University, Zhanjiang, Guangdong, China; Yunnan Technology and Business University, CHINA

## Abstract

Fulfilling corporate social responsibility (CSR) is crucial for society’s sustainable development. Executives’ academic experience significantly affects their awareness of social responsibility, value orientation, professional ability, and network resources. Thus, it is critical in CSR-related corporate decision-making. This paper explores the impact of executives’ academic experience on the fulfillment of CSR. It focuses on non-financial, Special treatment enterprises (ST), and ST* enterprises listed in the A-share market from 2012 to 2021. It utilizes a fixed-effects analysis model to examine the relationship between executives’ academic experience and CSR fulfillment. The CSR score and executives’ academic experience were positively correlated. This paper also explores the intermediary role of compensation incentives and the moderating effect of marketization level. Both compensation incentives and the level of marketization positively moderated the relationship between executives’ academic experience and CSR fulfillment. Meanwhile, the robustness results showed that the experimental findings still held after replacing the explained and explanatory variables. This paper contributes to the advancement of the Upper Echelons Theory and provides empirical evidence for the society’s sustainable development.

## 1. Introduction

Corporate social responsibility (CSR) implies that in addition to fulfilling its economic responsibilities, an enterprise must undertake the corresponding responsibilities toward the relevant investors, internal employees, and other stakeholders; the enterprise should also shoulder ethical and charitable responsibilities toward society, the environment, the upstream and downstream enterprises, the purchasers, and other relevant groups in course of its daily production and operation [1]. The fulfillment of social responsibility by enterprises is an important means of business management, which can not only provide help for the harmonious development of society but also help enterprises obtain higher consumer support, reduce market risks, establish a good corporate image, and enhance corporate strength [2,3]. It promotes the harmonious unity of the enterprise, ecology, nature, and society, thus facilitating sustainable development.

Existing literature indicates that various factors influence an enterprise’s sustainable development [4,5]. For instance, shareholders can contribute to the enterprise’s sustainable performance improvement when they effectively fulfill their supervisory role of effective supervision again [6]. Family business helps firms to innovate [7] and improve performance [8], thus promoting the enterprise’s sustainable development. Additionally, the enterprise can promote green innovation through the equity incentive of the executives to increase profits [9], and employee equity incentives can also effectively reduce costs and improve performance [10]. It has been argued that implementing external incentives can help reduce costs and improve performance. Several studies also suggest that effective external auditing can help firms be sustainable. However, Naoki Watanabel’s study yielded the opposite results [11]. Therefore, further research is needed to determine whether executives’ academic experience can contribute to the fulfillment of CSR and, consequently, to the enterprise’s sustainable development.

According to the Upper Echelon Theory, the educational level, professional background, economic status, personal experience, and other characteristics of corporate executives affect an enterprise’s corporate governance, investment decisions, and business performance. Presently, research on the influence of executives’ characteristics on the fulfillment of CSR focuses more on executives’ educational background, professional background, personal experience [12], career background [13], risk appetite [14], tenure [15], whether they work in the industry [16], and so on. The focus is less on the influence of executives’ academic experience. Hence, this paper considers executives’ academic experience as an entry point to explore its influence on CSR.

In terms of policy, the national “14th Five-Year Plan” proposes to encourage enterprises to engage in technological innovation and establish a system [5]. Based on the Opinions on Deepening the Reform of Talent Development System and Mechanisms [17], provinces and cities issued policies to encourage university teachers and researchers to work part-time in an appropriate manner. With the introduction of the policy, an increasing number of highly educated and talented individuals are joining enterprises to not only become the enterprise’s backbone but also provide intellectual assistance to its high-quality and sustainable development. Thus, it is practically significant to explore the influence of executives with academic experience on the fulfillment of CSR.

Several studies focus on the impact of executives with academic experience on the firm. In existing research, it has been found that not only the quality of corporate auditing can effectively regulate corporate ESG [18], executive characteristics can also effectively improve corporate CSR performance [19], improve the level of corporate environmental performance [20,21] and promote environmentally sustainable development [22]. Tax avoidance behavior of enterprises can also be reduced by enhancing the executives’ sense of social responsibility and increasing their risk avoidance awareness through academic experience [23]. Additionally, scholars found that executives’ academic experience significantly affects the cost of corporate debt financing, corporate innovation, CSR [24], corporate innovation [25], dividends [26], share price collapse [27], cash dividend policy [28], performance [29], and so on. Although some scholars already found that executives’ academic experience impacts the fulfillment of CSR by enterprises [30–32], fewer scholars studied executive academic experience, executive compensation, enterprise marketization level, and CSR simultaneously and explored the mechanism of the role of the aforementioned factors.

To synthesize the aforementioned analysis, this paper considers executives’ academic experience as an entry point as well as considers the non-financial, ST*, and ST enterprises in the A-share listed companies from 2012–2021. It utilizes databases, such as CSMAR, wind, Xunxun.com, and China’s sub-province marketization index. Resumé information of executives of the listed enterprises was manually collected to examine whether executives’ academic experience, based on their enterprise’s CSR disclosure, affects CSR fulfillment. This paper further examines the mediating effect of compensation incentives on academic experience and CSR to explore the influence mechanism. It also explores its moderating role in the mediating effect of compensation incentives based on the marketization level.

The three main contributions of this paper are as follows:

First, this study examines the influence of executives’ academic background on CSR fulfillment using the Imprinting Theory framework. This paper expands the understanding of how executives’ characteristics affect not only corporate performance but also social performance, thereby contributing to the existing literature on the impact of executives’ characteristics on social performance.

Second, we analyze the mediating role of compensation incentives between executives’ academic experience and CSR from the perspective of compensation incentives. Additionally, we examine the moderating effect of compensation incentives on marketization levels. This paper contributes to the advancement of the Upper Echelon Theory.

Third, by analysing the impact of corporate executives’ academic experience, corporate compensation incentives and marketisation level on corporate social responsibility, on the one hand, the research on top echelon theory adds theoretical value; on the other hand, for the government, it can promote more academics with academic experience to take part-time jobs in corporations through policy guidance to promote the upgrading of China’s economy. At the same time, enterprises can actively respond to the government’s call to take advantage of the policy to promote enterprise innovation and sustainable development.

## 2. Literature review and research hypothesis

### 2.1 Executive academic experience and CSR

The Upper Echelon Theory posits that the characteristics of the executives themselves influence corporate decisions [33]. The Imprinting Theory states that a subject will be “imprinted” by the characteristics of the environment at a particular time and that this “imprint” will have a long-lasting effect on the subject’s behavior [34]. Executives with academic experience, which plays a crucial role in their career development, are inevitably influenced in various ways. This influence can have an “imprinting” effect on their future career trajectory, ultimately impacting corporate decision-making and CSR. This specifically manifests in the following two aspects:

First, compared to other executives, those with academic experience have a stronger sense of social responsibility and self-restraint. Academic work can positively impact staff members due to the unique working environment and professional expectations. It can foster a heightened sense of social service, moral values, and integrity consciousness. For instance, colleges and universities often have higher moral and ethical expectations of their teachers. Society has bestowed a higher social status upon academic workers. Consequently, academic workers feel compelled to maintain a professional image, leading them to exercise greater self-restraint. This is done to contribute to society and enhance personal moral standards and social responsibility [35]. Therefore, executives with academic experience are more likely to have a higher social status. Consequently, executives with academic experience will actively fulfill their social responsibilities due to their previous work experience and the need to uphold their personal reputation and social status.

Second, executives with academic experience have multiple advantages in terms of professional and social resources. On the one hand, academic experience as a special trait has higher requirements on the professional level of practitioners, and the high level of professional and technical ability helps executives harness their ability to assess the enterprise’s innovation and offer theoretical assistance for corporate decision-making [36]. On the other hand, executives can utilize their expertise in the field of corporate innovation. Executives can also make use of the network of contacts they mastered during their academic work to understand the latest technological changes in the industry more quickly, reduce the negative impact of information asymmetry, improve the accuracy of decision-making, and thus help enterprises obtain more help from investors and their cooperation. Therefore, executives with academic experience can utilize their professional ability and social relationship resources to strive for more developmental advantages for the enterprise and promote its sustainable development; the enterprise’s development and growth are also conducive to improving its enthusiasm in fulfilling its social responsibility.

To summarize, this paper concludes that executives’ academic experience can help enterprises improve their ability to fulfill their social responsibility. Accordingly, we propose the following hypothesis H1:

H1: Executives’ academic experience contributes to CSR and has a significant positive effect on social responsibility.

### 2.2 Executives’ academic experience and compensation incentives

Executives’ compensation generally comprises both monetary and long-term incentives. Monetary compensation includes salaries and bonuses, while long-term incentives include stock or options. The salary is fixed and has nothing to do with the performance of the executive in the same month or quarter; the bonus is generally the incentive issued by the enterprise according to the assessment of the executive’s ability and performance. The long-term incentive compensation, due to its deferred nature, can realize the role of the executive and the enterprise to prosper together [37]. The long-term incentive compensation can realize the role of the executive and the enterprise as one. Compared to executives without academic experience, those with academic experience can utilize their professional ability, social resources, information gap, and other advantages to promote the enterprise’s innovation and win investment. Therefore, to obtain the “resources” of executives with academic experience and promote the enterprise’s sustainable development and social influence, its controlling shareholders may obtain a higher level of remuneration incentives for executives with academic experience compared to those without academic experience. According to Maslow’s Hierarchy of Needs Theory, it is evident that a high remuneration for listed executives can fulfill their high-level needs, such as pursuing personal ambitions and gaining a social reputation. This aligns with the executives’ “imprint of academic experience.”

In existing studies, scholars have found that the controlling shareholders of an enterprise wishing to intervene in corporate strategy or decision-making will necessarily need to rely on executives [38]. The academic experience of executives brings resources to the enterprise, but they need to bear the agency problem brought by the controlling shareholders. While executives with academic experience bring valuable resources to the enterprise, they also face the challenge of the agency problem posed by the controlling shareholders. To align with the goals of these shareholders, the executives must be provided with risk compensation and high levels of incentive compensation to mitigate or eliminate the agency problem. Therefore, hypothesis H2 is proposed as follows:

H2: Executives with academic experience receive higher levels of pay incentives and have a significantly positive effect on the level of pay incentives compared to executives without academic experience.

### 2.3 Pay incentives and social responsibility

The Upper Echelon Theory established that executive traits, compensation incentives, and CSR interact with each other [33]. Most scholars so far believe that offering high levels of compensation incentives can assist enterprises in fulfilling their social responsibility more effectively. Zhao Lu’s study found that CEOs with high levels of compensation have a higher level of fulfillment of their CSR [39]. Some studies also found that fulfilling CSR obligations is linked to executive compensation. In other words, when an enterprise achieves a high level of CSR fulfillment, it tends to correspond to higher levels of executive compensation [40]. While there may be a relationship between executive compensation and CSR performance, this relationship is influenced by the level of competition in the industry. Additionally, CSR performance is also influenced by the level of competition in the industry [41]. CSR performance varies depending on the level of monetary compensation and equity incentives in the compensation package.

The Contract Theory suggests that the separation of ownership in enterprises can result in managers who prioritize their control over maximizing shareholders’ interests. This misalignment of interests between shareholders and managers creates a phenomenon called “agency conflict,” where the business objectives of the owners and managers diverge. Jia Xianfeng argued that agency costs have a positive effect on promoting compensation incentives and the fulfillment of CSR [42]. Therefore, implementing high-level compensation incentives for executives, on the one hand, can effectively reduce the agency problem, mitigate the conflict between corporate owners and managers, and align their interests. On the other hand, it can also motivate the executives to actively fulfill their job responsibilities, establish a positive corporate image, and enhance corporate performance and social responsibility, thereby improving the CSR score. In summary, hypothesis H3 is proposed as follows:

H3: Executive compensation incentives promote active CSR.

### 2.4 The mediating role of executive compensation incentives

For executives with academic experience, the compensation incentive mechanism can play an intermediary role in the impact of CSR. A well-designed compensation incentive mechanism can not only meet the executives’ basic needs but also help them pursue higher-level psychological needs. It can also facilitate the integration of the executives’ personal image with the corporate image. On the one hand, a positive corporate image can enhance the company’s reputation and increase its market competitiveness. On the other hand, it can contribute toward building the executives’ high-quality personal image. This in turn helps them gain social recognition and reputation. Thus, reasonable compensation incentives can effectively improve work efficiency, promote the enhancement of corporate and social performance, and encourage enterprises to more actively fulfill their social responsibilities. Moreover, such incentives can facilitate the alignment of interests between executives and shareholders. Executives will be motivated to prioritize the interests of shareholders as they pursue the business objectives. This approach serves as a business strategy for social responsibility, as it not only enhances the enterprise’s reputation but also improves labor relations and secures a long-term workforce for it. Therefore, executives who share common interests with shareholders will more effectively enhance the level of CSR fulfillment. On the contrary, if the enterprise’s compensation incentive policy is imperfect or fails to meet the executives’ basic needs, they will naturally prioritize investing the enterprise’s resources into its development to improve financial performance while neglecting their social responsibilities. Based on this, we propose the following hypothesis H4:

H4: Compensation incentives mediate the relationship between executives with academic experience and CSR fulfillment.

### 2.5 The moderating role of marketization level

Executives’ academic experience may be motivated by financial incentives to fulfill CSR, and the way it works may be influenced by marketization. The fulfillment of CSR may vary depending on the economic level of the enterprise, which can be influenced by the level of marketization in the region where the enterprise is situated. Therefore, when executives make economic decisions, they not only need to consider the enterprise’s internal environment but also the influence of the external market environment on decision-making. When the enterprise operates in a highly marketized environment, the “academic imprint” instills executives with a greater sense of social responsibility and awareness of social service. Additionally, it enhances their reputation, enabling executives to make decisions that more effectively drive the enterprise to actively fulfill its social responsibility. At the same time, in regions with a high marketization level, where policy mechanisms, business environment, and information symmetry are more advantageous, market competition becomes more intense. Executives with academic experience possess not only the “academic identity” that other executives lack but also a high level of professional competence and social resources. These factors help the enterprise stand out in the fiercely competitive market. Therefore, enterprises adopt salary incentives to improve the compensation of executives who possess academic experience. This helps establish a positive corporate image through the executives’ “academic identity” and facilitates further collaboration with investors, consumers, and the government. Executives’ high-level professional abilities and social resources heighten the competitiveness of enterprises, leading to improved cumulative performance and social impact. Consequently, this promotes the overall development of enterprise performance and encourages enterprises to actively fulfill their social responsibilities.

Based on this analysis, we propose hypothesis H5:

H5: The marketisation level positively moderates the mediating role played by executive compensation incentives in the impact of executive academic experience on corporate social responsibility.

## 3. Research design

### 3.1 Sample and data

This study selects non-financial, ST, *ST enterprises in A-share listed enterprises from 2012–2021 with as the research object, draws on the existing literature practice to deal with the sample of some enterprises in the research object, the processing criteria are as follows: (1) the enterprises with serious missing main data are excluded; (2) for the sake of observability of the results, the data is standardised uniformly; (3) for the sake of excluding the extreme data’s influence on the research results, this study adopts generalised treatment for the data. The total number of valid sample data in this study is 14,586, and the relevant data in this study come from Hexun.com, China Provincial Marketisation Index Database, Cathay Pacific (CSMAR), and Wind Database.

### 3.2 Definition of variables

**Fig 1:**
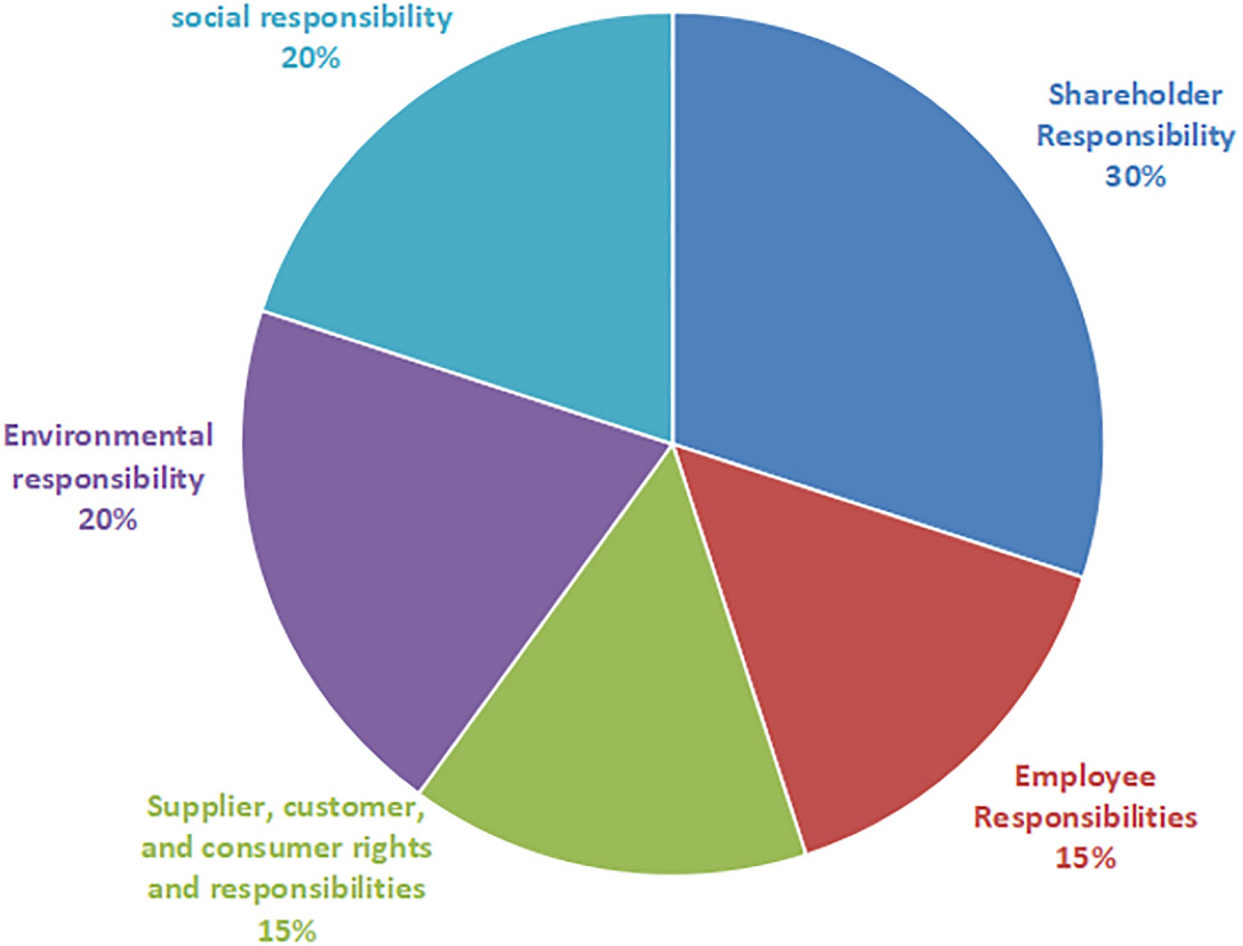
Ratio of corporate social responsibility scores on Hexun.com.

1. Explanatory variables. The main explanatory variable in this paper is executives’ academic experience (Aca-exp), which was obtained from the corporate executive information disclosed in the Cathay Pacific database. This paper draws on the research conducted by Kai-Tang Zhou [43]. It assigned the value of Aca-exp as a dummy variable to measure the executives’ academic experience. Aca-exp was set to 1 if one or more of the CEO, board of directors and supervisors, legal representatives, and senior executives directly involved in the enterprise management have worked or are currently working in higher education institutions, scientific research institutes, or industry associations. It was set to 0 if the opposite is true [44]. This paper used Aca-exp1 to measure the proportion of executives with academic experience in the executive team.
2. Dependent variables. CSR is the explanatory variable, with its main data source being Hexun.com. Since the social responsibility scores published by Hexun.com are considered to be objective, comprehensive, and highly credible, we used these scores as a criterion to measure the level of fulfillment of social responsibility by enterprises. The social responsibility scores and the level of social responsibility fulfillment by enterprises were positively correlated.The social responsibility score of Hexun.com includes the following five aspects: shareholder responsibility, employee responsibility, supplier, customer and consumer rights responsibility, environmental responsibility, and social responsibility. The specific rating is shown in Fig 1.
3. Mediating variable. In the measurement of the mediating variable (executive compensation incentives) mainly refers to the research of Zhang Guofu [16], Kong Feng [37], Zhao Lu [39], Xu Ailing [40], and other scholars, and conducts an empirical study by manually collecting executive compensation disclosed by the financial reports of each enterprise, accounting for the sum of the compensation of the executives whose compensation ranked in the top three, and at the same time, taking the natural logarithm of the sum of the compensation.
4. Moderating variable. The moderating variable in this study is the marketisation level, and the empirical study analyses whether different regions affect corporate executive compensation due to different levels of marketisation. The marketisation index is used to measure the regional marketisation level, and the data of the marketisation index is obtained from the database of China’s marketisation index by province.
5. Control variables. Referring to the existing literature, this paper selected the following control variables: company size (Size), executive team size (Board), duality (Board structure), corporate profitability level (Roa), and gearing ratio (Lev). To enhance the validity of the findings, we included two dummy variables for industry (Ind) and year (Year). See the Appendix for variable definitions.

### 3.3 Regression models

To test hypotheses H1, H2, H3, and H4, the regression models (1, 2, 3, and 4) were set up:

CSRit=a0+a1Aca-expit+a2Controlsit+ΣInd+ΣYear+εit (1)

Salaryit=b0+b1Aca-expit+b2Controlsit+ΣInd+ΣYear+εit (2)

CSRit=c0+c1Salaryit+c2Controlsit+ΣInd+ΣYear+εit (3)

CSRit=d0+d1Aca-expit+d2Salaryit+d3Controlsit+ΣInd+ΣYear+εit (4)

Among them, Model (1) was used to test the impact of the executives’ academic experience on CSR fulfillment. If a1 was significantly negative, this indicated that executives’ academic experience had an inhibitory effect on CSR fulfillment. If it was significantly positive, this suggested a positive promotional effect, thus supporting hypothesis H1. Model (2) tested the impact of executives’ academic experience on their compensation. If b1 was significantly positive, this indicated that executives’ academic experience had a positive effect on their compensation, supporting hypothesis H2. Conversely, if b1 was significantly negative, it suggested an inhibitory effect. Model (3) tested the impact of executive compensation on CSR. If c1 was significantly positive, this indicated that executive compensation incentives played a positive role in promoting CSR, supporting hypothesis H3. Conversely, if c1 had a negative effect, this suggested an inhibitory effect. Model (4) tested the mediating role of compensation incentives between executive academic experience and CSR.The mediating role of model (4) is explained in detail as follows: the mediating role of corporate compensation incentives exists in the following two cases, the first one is if the coefficient of the executive academic experience d1 and the coefficient of the corporate compensation incentives in the model (4) are greater than 0 and significant Firstly, if the coefficients of d1 and d2 in model (4) are both greater than 0 and significant, and the coefficient of executive academic experience a1 (from model 1) > d1, then it can be shown that corporate pay incentives play a partially mediating role in the relationship between executive academic experience and CSR; secondly, if d2 in model (4) is greater than 0 and significant, but d1 is not significant, then it can be shown that corporate pay incentives play a fully mediating role in the relationship between executive academic experience and CSR. Both of these possibilities justify the hypothesis.

To investigate the moderating impact of marketization level on executives’ academic experience and CSR, we included the cross-multiplier terms of executive academic experience, marketization level, and their relationship. Model (1) proposed by Wen Zhonglin et al. [45] served as the basis for this analysis. Additionally, we included the cross-multiplier terms of executive academic experience, marketization level, and its relationship with executive academic experience based on Model (1); we added the cross-multiplier terms of compensation incentives and marketization level to determine the moderating effect of marketization level on compensation incentives in executive academic experience and CSR performance. If the coefficient of the cross-multiplier term was positive, this indicated that the level of marketization positively influenced the pay incentives in the relationship between executives’ academic experience and the fulfillment of CSR, validating hypothesis H5. Conversely, if the coefficient of the cross-multiplier term was negative, hypothesis H5 stood invalid. Construct models (5 and 6) as follows:

$$\begin{array}{l} {\text{Salary}}_{\text{it}}={\text{e}}_{0}+{\text{e}}_{1}\;{\text{Aca-exp}}_{\text{it}}+{\text{e}}_{2}\;{\text{Market}}_{\text{it}}+{\text{e}}_{3}\;{\text{Market}}_{\text{it}}*{\text{Aca-exp}}_{\text{it}}+\\ {\text{e}}_{4}\;{\text{Controls}}_{\text{it}}+\Sigma \text{Ind}+\Sigma \text{Year}+\varepsilon_{\text{it}} \end{array}$$ (5)

$$\begin{array}{l} {\text{CSR}}_{\text{it}}={\text{f}}_{0}+{\text{f}}_{1}\;{\text{Aca-exp}}_{\text{it}}+{\text{f}}_{2}\;{\text{Market}}_{\text{it}}+{\text{f}}_{3}\;{\text{Salary}}_{\text{it}}+{\text{f}}_{4}\;{\text{Salary}}_{\text{it}}*{\text{Market}}_{\text{it}}+\\ {\text{e}}_{4}\;{\text{Controls}}_{\text{it}}+\Sigma \text{Ind}+\Sigma \text{Year}+\varepsilon_{\text{it}} \end{array}$$ (6)

## 4. Empirical results and analysis

### 4.1 Descriptive statistics

The descriptive statistics in Table 1 Descriptive statistics indicate that the sample enterprises have a standard deviation of 16.279, a maximum value of 78.895, and a minimum value of -2.177 for CSR. This suggests that some of the listed enterprises in the sample do not prioritize fulfilling their social responsibility.

**Table 1:** Descriptive statistics.

Variant	N	Average value	Standard deviation	Maximum values	Upper quartile	Minimum value
CSR	14586	25.015	16.279	78.895	22.349	-2.177
Aca-exp	14586	0.359	0.479	1.000	0.000	0.000
Aca-exp1	14586	0.085	0.142	1.000	0.000	0.000
Salary	14586	13.989	0.652	16.101	14.255	12.573
Market	14586	7.899	1.868	11.400	-1.423	8.100
Size	14586	22.151	1.189	26.519	21.861	19.005
Board	14586	6.431	2.025	16.000	5.000	3.000
Duality	14586	0.239	0.418	1.000	0.000	0.000
Roa	14586	0.0381	0.0529	0.195	0.033	-0.218
Lev	14586	0.445	0.223	0.928	0.411	0.049

Additionally, the fulfillment of social responsibility among China’s listed enterprises is widely dispersed, with significant variations in their level of commitment. Furthermore, the median value for social responsibility is only 22.349, indicating that there is still room for improvement in the fulfillment of social responsibility by these enterprises. The average value of executives with academic experience in enterprises is 0.359. Additionally, the average value of the ratio of executives with academic experience to the total number of executives in the executive team is 0.085. These figures indicate that it is not uncommon for high-level talents with academic experience to serve as executives in China’s listed enterprises. The analysis of executive compensation highlights a significant disparity between the highest and lowest values of compensation. The median and average values, however, accurately represent the overall magnitude of executive compensation. The data related to the marketization level indicate a significant disparity between the maximum value (11.400) and the minimum value (-1.423). This highlights a significant degree of variation between the sample enterprises, suggesting that the issue of uneven regional development in China remains prominent. Table 2 shows the descriptive statistics of executive "Aca exp" with academic experience.

**Table 2:** Descriptive statistics for the variable academic experience “Aca-exp”.

Variable	Number of obs = 0	%	Number of obs. = 1	%
Aca-exp	9354	64.1%	5232	35.9%

### 4.2 Correlation analysis

Pearson correlation coefficient analysis was used to determine the influence between the variables. First, according to the results of Table 3, the correlation coefficient of executives’ academic experience on CSR is 0.038, which has a positive promoting effect, and it is significant at a 5% level, and this result can preliminarily affirm H1. Second, the coefficients of executive compensation incentives on the fulfillment of CSR and executives’ academic experience are 0.211 and 0.058, respectively. Both coefficients are significant at the 1% confidence level. This suggests that corporate compensation incentives have a positive relationship with the fulfillment of CSR and also have a positive impact on improving the level of CSR. It indicates that corporate compensation incentives are positively correlated with executives’ academic experience. Additionally, it has a positive effect on the improvement of the level of CSR, thus preliminarily affirming hypotheses H2 and H3.

**Table 3:** Analysis of variable correlation coefficients.

**变量**	**CSR**	**Aca-exp**	**Aca-exp1**	**Salary**	**Market**	**Size**	**Board**	**Duality**	**Roa**	**Lev**
**CSR**	1									
**Aca-exp**	0.038**	1								
**Aca-exp1**	0.019	0.726*	1							
**Salary**	0.211***	0.058***	0.013*	1						
**Market**	0.038*	0.119***	0.009***	0.140***	1					
**Size**	0.195***	0.021***	0.003***	0.401***	0.148***	1				
**Board**	0.139***	0.071***	-0.068**	0.143**	0.121***	0.215***	1			
**Duality**	0.041**	-0.109***	-0.101***	-0.015	0.201***	-0.159***	0.070***	1		
**Roa**	0.275***	-0.002	-0.001	0.170***	0.152**	-0.008*	0.033*	0.028**	1	
**Lev**	-0.101**	-0.001	-0.001	0.107*	-0.103*	0.413**	0.072*	-0.173***	-0.451**	1

Note:

***, **, and * denote significant at the 1%, 5%, and 10% levels, respectively, and the same below.

Please see the Appendix for variable abbreviations.

### 4.3 Regression analysis

#### 4.3.1 Main regression analysis and mediation test

Table 4 presents the regression results of the benchmark regression and the mediating role. When no other variables are involved, the regression coefficient of executives’ academic experience on CSR is 1.895. This coefficient is significant at the 1% confidence interval, suggesting that executives’ academic experience plays a positive role in promoting CSR. As can be seen in column (2), without considering the mediating effect of salary incentives, it is significant at a 1% confidence interval, which indicates that executives’ academic experience can promote the level of CSR. Therefore, hypothesis H1 stands affirmed. The positive impact of academic experience of executives on corporate social responsibility may be due to two reasons: firstly, academic experience may effectively enhance the sense of responsibility and self-restraint of executives. For example, some executives may have worked as teachers in higher education institutions, and their teacher status allows executives to actively promote corporate social responsibility in order to maintain their personal reputation and social status. Secondly, academic experience may effectively enhance the social service awareness, moral level, and integrity awareness of executives, thereby having a positive impact on the fulfillment of corporate social responsibility. The regression results in column (3) indicate that the regression coefficient of executives’ academic experience and compensation incentives is 0.913, which is significant at the 1% level. This implies that academic experience can enable executives to obtain higher compensation in the enterprise, affirming hypothesis H2. According to column (4), the regression coefficient of executive compensation and CSR is positive, and the regression coefficient is 1.701, which is significant at the 1% level. This shows that the higher the compensation incentives, the more will be the executives’ ability to positively contribute to the fulfillment of CSR. Thus, hypothesis H3 is affirmed. The regression results of column (5) indicate that executive compensation incentives can improve the CSR fulfillment ability. Further, the academic experience of executives in column (5) is significant at the 1% level, and the regression coefficient is 2.01, which is smaller than that of the regression coefficient of the academic experience of executives in column (2). According to Wen Zhonglin et al. [45], the empirical results indicate that the limitations of executives’ academic experience on the ability to actively promote CSR, the regression coefficients of academic experience, and compensation incentives are significant at the 1% level. Additionally, the regression coefficients of the above two variables are positive, indicating that some of the mediating role of compensation incentives is significant. This affirms hypothesis H4.

**Table 4:** Main regression analysis and mediation test.

Variant	CSR (1)	CSR (2)	Salary (3)	CSR (4)	CSR (5)
Aca-exp	1.895*** (3.11)	2.212*** (3.67)	0.913*** (5.88)		2.01*** (3.06)
Salary				1.701*** (7.03)	1.597*** (6.72)
Size		3.580*** (9.79)	0.207*** (29.31)	3.276*** (8.91)	2.215*** (5.92)
Board		0.445*** (2.79)	0.047*** (2.93)	0.032*** (2.82)	0.349** (2.46)
Duality		1.015 (1.49)	0.074*** (5.98)	-0.018*** (-2.79)	1.01 (1.40)
Roa		1.632*** (3.19)	0.625*** (3.41)	1.457*** (2.98)	1.608*** (8.16)
Lev		-10.751*** (-4.97)	0.045*** (3.61)	-11.803*** (5.15)	-13.13*** (-10.18)
Year	containment	containment	containment	containment	containment
Ind	containment	containment	containment	containment	containment
N	14586	14586	14586	14586	14586
R^2^	0.151	0.224	0.253	0.259	0.282

#### 4.3.2 The moderating role of marketization level

Table 5 presents the regression results of the moderating effect of the marketization level. In the regression results of compensation incentives, after adding the cross-multiplier term between executives’ academic experience and the marketization level, the regression coefficient of the former slightly reduced to 1.679, which is significant at the 1% level as in the benchmark regression. This indicates that the higher the marketization level, the more effective the effect of executives’ academic experience on compensation incentives. Meanwhile, the regression results of CSR show that the regression coefficient of compensation incentive is 2.275 and significant at 1% level, while the coefficient of the cross-multiplier term of compensation incentive and the level of marketization is 3.904, which is not significant. Hence, the moderating effect of the marketization level on the dynamic between compensation incentives in the context of executives’ academic experience and the fulfillment of CSR is established and positively promotes the effect. That is to say, compared to regions with a high marketization level, the promotion effect of academic experience on social responsibility is weaker in regions with a low marketization level. Therefore, hypothesis H5 stands affirmed.

**Table 5:** Regression results of the moderating effect of marketization level.

Variant	Salary	CSR
Aca-exp	0.158*** (9.17)	1.679*** (2.68)
Market	4.621*** (4.03)	-0.235 (-1.01)
Aca-exp *Market	1.416** (2.19)	
Salary		2.275*** (9.09)
Salary *Market		3.904 (0.45)
Size	0.896*** (11.82)	3.991*** (6.43)
Board	0.192* (1.79)	0.213** (2.10)
Duality	0.0387** (2.14)	-0.485 (-0.879)
Roa	0.790 (1.25)	1.422** (2.23)
Lev	-13.341*** (-10.01)	-14.119*** (-10.26)
Year	containment	containment
Ind	containment	containment
N	14586	14586
R^2^	0.113	0.097

### 4.4 Robustness tests

To strengthen the credibility of our research conclusions, in the robustness test, the explanatory variable of CSR score was replaced with the CSR grade of Hexun.com, and the explanatory variable of executives’ academic experience was replaced with the percentage of executives with academic experience; the regression was conducted again. Table 6 shows the results of the robustness test. It indicates the following: After replacing the explanatory and explanatory variables, executives’ academic experience can still significantly promote CSR positively; the mediating effect of compensation incentives is significant, and the marketization level still plays a moderating role in the mediating effect of compensation incentives after replacing the variables, which is in line with the aforementioned findings. This establishes that this paper’s conclusions have a certain degree of credibility.

**Table 6:** Robustness test.

variant	CSR1 (1)	CSR1 (2)	Salary (3)	CSR1 (4)	CSR1 (5)	Salsry (6)	CSR (7)
Aca-exp1	0.212** (1.97)	0.314*** (2.76)	0.673*** (4.89)		0.261** (2.25)	0.093*** (6.18)	0.338*** (3.01)
Salary				0.155*** (6.01)	0.072*** (4.63)		0.421*** (3.98)
Aca-exp *Market						0.301** (2.35)	
Salary *Market							0.576 (1.66)
Year	containment	containment	containment	containment	containment	containment	containment
Ind	containment	containment	containment	containment	containment	containment	containment
N	14586	14586	14586	14586	14586	14586	14586
R^2^	0.08	0.11	0.32	0.16	0.17	0.15	0.08

## 5. Conclusions and recommendations

### 5.1 Conclusion

This paper selected non-financial, state-owned (ST), and state-owned enterprises (ST) in A-share listed enterprises as research subjects from 2012 to 2021 to investigate the effects of executives’ academic experience, compensation incentives, and marketization level on CSR. It expands the Upper Echelon Theory, enriches the existing literature, and provides empirical evidence for the effect of executives’ academic experience on CSR fulfillment. The empirical results show that executives’ academic experience contributes to the fulfillment of CSR. Additionally, CSR scores and executives’ academic experience are positively related. Second, executive compensation incentives also contribute to the promotion of CSR. Third, compensation incentives mediate the relationship between executives’ academic experience and CSR fulfillment. Lastly, the marketization level positively moderates the mediating effect of compensation incentives. Meanwhile, the robustness results show that the aforementioned experimental conclusions still hold after replacing the dependent and independent variables. Therefore, empirical findings indicate that the academic experience of executives contributes to the fulfillment of CSR and positively affects society’s sustainable development.

### 5.2 Policy recommendations

Based on the results of the empirical tests, we offer the following recommendations:

1. The government should encourage universities, research institutes, and other highly educated units to allow high-level talents to work part-time in enterprises and entrepreneurship. This can be achieved by implementing policies that protect the welfare benefits of high-level talents, ensuring stability, and providing a sense of security for them to work. Highly educated individuals possess unique qualities that stem from their work experience, social resources, and professional abilities. These qualities contribute toward improving the accuracy of enterprise decision-making, thereby assisting enterprises in acquiring additional resources to promote sustainable development and enhance CSR.
2. For executives with academic experience to serve in enterprises, in addition to the government’s provision of protection policies, if the enterprises recognize them, they can also attract them to take root and build nests in the enterprises through scientific and reasonable remuneration incentive policies. This will improve the work motivation of executives with academic experience, ensure the steady improvement of their work efficiency, promote the economic benefits of the enterprises, and push forward the fulfillment of the social responsibilities of the enterprises.
3. The continuous improvement of the degree of CSR fulfillment also contributes to the healthy development of society. Although there is a national proposal to encourage enterprises to fulfill their social responsibility, and the State-owned Assets Supervision and Administration Commission (SASAC) has established a social responsibility bureau accordingly, there is currently no comprehensive social responsibility law. Therefore, the government should actively promote legislation on social responsibility, strengthen the external supervision of enterprise compliance, and increase consumer oversight. This two-pronged approach will enhance the level of social responsibility fulfillment by enterprises.

## 6. Analysis of limitations

Despite enriching the existing literature, this paper has limitations. First, it only adopts the data of listed companies in China, due to the differences in economic systems and social environments, which may make the influence of the executives’ characteristics on the fulfillment of CSR differ across countries. Second, this paper only utilizes data that have been disclosed by listed companies for analysis, restricting the research. In future research, questionnaires and case studies can be used to analyze the impact of executives’ characteristics on the fulfillment of CSR on a deeper level. Third, although this paper incorporates compensation incentives and marketization level into the research framework for discussion, there remain limitations. In the future, it would be beneficial to include additional moderating factors that affect CSR fulfillment, such as political and economic system factors, in the research framework. It is also necessary to use more countries’ samples for analysis in future research.

## Supporting information

S1 Appendix Definition of variables. (DOCX)

S1 Data (XLSX)
