# Supplementary material for: Executives’ academic experience and corporate social responsibility: A case study in China
Source: PLoS One. 2024 Jun 25;19(6):e0305813. doi: 10.1371/journal.pone.0305813 (PMC11198777; doi:10.1371/journal.pone.0305813)
Supplement: S1 Appendix — (DOCX) [file pone.0305813.s001.docx]

Appendix:

Definition of variables

| Variable category | Variable name | Variable symbol | Variable definition |
| --- | --- | --- | --- |
| Explanatory variable | Corporate social responsibility | CSR | CSR score |
| Explanatory variable | Executive academic experience | Aca-exp | Dummy variable, which was assigned a value of 1 if one of the executives in the enterprise had academic experience, and 0 if the opposite was true |
|  | Percentage of executives with academic experience | Aca-exp1 | Number of executives with academic experience as a ratio of total executive team size |
| Intermediary variable | Executive compensation incentives | Salary | Natural logarithm of the sum of the top three executive compensation rankings |
| Moderator variable | Level of marketization | Market | Measured by the marketization index of the enterprise’s location |
| Control variable | Enterprise size | Size | Take the natural logarithm of the assets of the listed enterprise, |
|  | Executive team size | Board | i.e., the total number of executive team members |
|  | Board structure | Duality | Dummy variable, assigned a value of 1 if the chairman of the board and the general manager were the same person, and 0 otherwise |
|  | Corporate profitability  Level | Roa | Net profit to total assets |
|  | Gearing | Lev | Ratio of total liabilities to total assets of the enterprise |
|  | Sector | Ind | Industry dummy variables |
|  | Year | Year | Year dummy variable |
